# Peripapillary Vessel Density in Eyes with Rhegmatogenous Retinal Detachment after Pars Plana Vitrectomy

**DOI:** 10.1155/2021/6621820

**Published:** 2021-03-23

**Authors:** Bing Lu, Pengfei Zhang, Haiyun Liu, Huixun Jia, Yang Yu, Fenghua Wang, Hong Wang, Xiaodong Sun

**Affiliations:** ^1^Department of Ophthalmology, Shanghai General Hospital (Shanghai First People's Hospital), Shanghai Jiao Tong University School of Medicine, Shanghai, China; ^2^Shanghai Key Laboratory of Ocular Fundus Diseases, Shanghai, China; ^3^National Clinical Research Center for Eye Diseases, Shanghai, China; ^4^Department of Ophthalmology, The First Affiliated Hospital of Wannan Medical College, Wuhu, China; ^5^Shanghai Engineering Center for Precise Diagnosis and Treatment of Eye Diseases, Shanghai, China; ^6^Shanghai Engineering Center for Visual Science and Photomedicine, Shanghai, China

## Abstract

**Purpose:**

To investigate the vascular density of the optic nerve head (ONH) and macula using optical coherence tomography angiography (OCTA) in patients undergoing vitrectomy for rhegmatogenous retinal detachment (RRD) and to evaluate associations with visual outcomes.

**Methods:**

Patients with RRD, successfully treated with a pars plana vitrectomy (PPV) and a minimum three-month follow-up were included in this study. The vessel density (VD) of the ONH and peripapillary regions, foveal avascular zone (FAZ), foveal flow density (FFD), and parafoveal flow density (PFD) were evaluated using OCTA and compared to the fellow eye. Multivariate linear regression analysis was performed to determine correlations with visual outcomes.

**Results:**

Thirty-one patients with macula-off RRD were included in the study. Compared with the fellow eyes, eyes after RRD surgery had a lower peripapillary VD (*P* < 0.01). No significant difference in superficial and deep FFD, PFD, and FAZ area was found compared to the fellow eyes. Postoperative peripapillary VD and baseline BCVA were significantly associated with BCVA three months after PPV (*P* < 0.05).

**Conclusion:**

Rhegmatogenous retinal detachment eyes successfully treated with PPV had lower peripapillary vessel density than fellow healthy eyes. Postoperative BCVA was related to postoperative peripapillary VD.

## 1. Introduction

Rhegmatogenous retinal detachment (RRD) is a vision-threatening disease characterized by separation of the sensory retina from the retinal pigment epithelium (RPE) [1]. Scleral buckling surgery and pars plana vitrectomy (PPV) with gas or silicone oil (SO) tamponade can be performed to reattach the retina [2, 3]. Despite the high anatomical restoration after vitreoretinal surgery, some patients continue to have poor visual recovery [4].

Some observations suggest that damage to the outer retinal layer significantly affects vision in eyes with RRD. Disruptions of the ellipsoid zone (EZ) and cone interdigitation zone (IZ) of the photoreceptors on spectral-domain optical coherence tomography (SD-OCT) are correlated with best corrected visual acuity (BCVA) after surgery [5, 6]. Other studies have focused on the decrease of the inner retina layer considered to be a poor prognostic factor after RRD surgery [7]. These results suggest that the recovery of retinal function after retinal reattachment depends on the status of both the inner and outer retinal layers.

The retinal microvasculature is the important component of the retinal structure and was reported to be involved in the development and recovery from several retinal diseases, including retinal detachment and diabetic retinopathy [8, 9]. Optical coherence tomography angiography (OCTA) is a novel imaging platform that enables closer observation of the blood flow of each retinal capillary layer in the macular and peripapillary regions [10–12]. Recent studies using OCTA demonstrated the augmentation of foveal avascular zone (FAZ) and capillary plexus flow density in the macular region after vitrectomy with gas tamponade in patients with RRD [9, 13]. However, these studies captured only the macular microvasculature; the changes in postoperative peripapillary vasculature and relationships between postoperative retina vasculature and visual outcome have not yet been investigated.

Hence, we used OCTA to investigate both the macular and peripapillary vasculature after PPV in patients with macula-off RRD and evaluated the associations with retinal vascular structure and postoperative visual outcomes.

## 2. Methods

This observational study was conducted in accordance with the ethical standards stated in the Declaration of Helsinki and with the approval of the Ethical Review Committee of Shanghai General Hospital, Shanghai Jiao Tong University School of Medicine. Patients treated for primary macula-off RRD at the Department of Ophthalmology, Shanghai General Hospital, between January 2018 and June 2019 were included. Our study included proliferative vitreoretinopathy (PVR) grade A or grade B [14]. The exclusion criteria were (1) PVR grade C or worse; (2) vitreous hemorrhage after PPV; (3) secondary epiretinal membrane (ERM), bilateral ERM, or macular hole; (4) recurrent retinal detachment after the initial PPV; (5) history of diabetic retinopathy, glaucoma, high myopia (greater than –6.0 diopters), anisometropia >2.0 diopters, or another ocular pathology that could interfere with visual function; (6) persistent subretinal fluid, intraretinal fluid, or disruption of retinal layers on OCT, in particular disruptions of EZ; (7) age <18 years; and (8) poor imaging data in OCTA (i.e., due to media opacity and eye movement).

Intraocular surgeries were performed by the same experienced surgeon (HW) using a 25G pars plana vitrectomy (Alcon Constellation Vision System; Alcon Laboratories Inc.). Intravitreal triamcinolone acetonide was used at the surgeon's discretion and none of the eyes underwent peeling of the inner limiting membrane (ILM). Air was used for gas tamponade and Oxane 5700 for SO tamponade (Bausch & Lomb, Inc.).

Before surgery, all patients underwent a comprehensive ophthalmic examination, including a BCVA measurement, slit-lamp examination, intraocular pressure (IOP) measurement (by Goldmann applanation tonometry) and noncycloplegic refraction (AR-310A, Nidek, Inc.), and dilated examinations with SD-OCT (Spectralis, Heidelberg Engineering, Inc.). Refraction data were converted to spherical equivalents (SE) using the spherical diopter (D) plus one-half of the cylindrical dioptric power. Three months after surgery, BCVA, IOP, slit-lamp examination, dilated fundus examination, SD-OCT, and OCTA measurements of bilateral eyes were performed.

**Figure 1:**
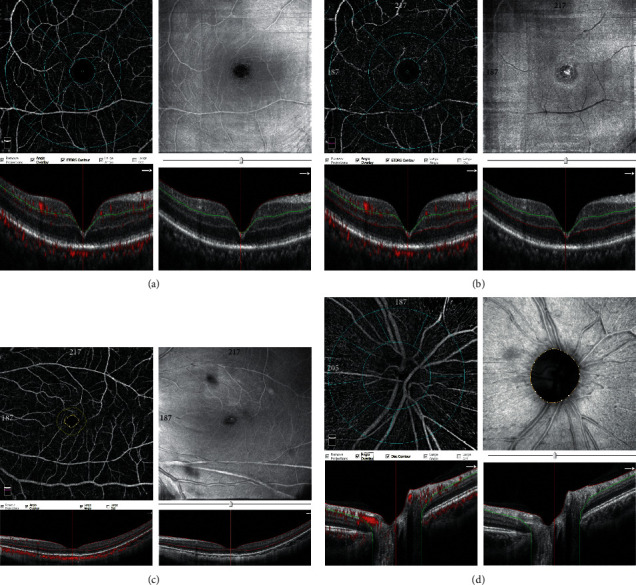
OCTA images with Angio Retina and Angio Disc mode. (a) Superficial foveal flow density (FFD) and parafoveal flow density (PFD): above, 6 × 6 mm en face angiogram; below, B scan image (red line = internal limiting membrane (ILM); green line = inner plexiform layer (IPL)). (b) Deep foveal flow density (FFD) and parafoveal flow density (PFD): above, 6 × 6 mm en face angiogram; below, B scan image (green line = IPL; red line = outer plexiform layer (OPL)). (c) Foveal avascular zone (FAZ): area of the inner yellow circle. (d) Vessel density (VD) of optic nerve head (ONH) and peripapillary region: above, 6 × 6 mm en face angiogram; below, B scan image (red line = ILM; green line = nerve fiber layer (NFL)).

OCTA was performed using an RTVue XR OCT Avanti System (software version 2018.1.0.43). The Angio Retina mode with a scanning area of 6 × 6 mm was used to measure foveal flow density (FFD) and parafoveal flow density (PFD). The flow density was calculated as the percentage of area occupied by vessels in the selected region. The FFD and PFD were defined as the flow density in the foveal region with a diameter of 1 mm and the parafoveal region with a diameter from 1 mm to 2.5 mm, respectively [15] (Figures 1(a) and 1(b)). The size of FAZ was assessed by two independent observers (BL and HW) (Figure 1(c)). The Angio disc mode with a scanning area of 4.5 × 4.5 mm was used to measure vessel density (VD) of the optic nerve head (ONH) and peripapillary region. The ONH and peripapillary VD were defined as the vessel density in the optic disc with a diameter of 2 mm and in the peripapillary region with a diameter from 2 mm to 4 mm, respectively [16]. The peripapillary VD values of the two peripapillary sectors (superior and inferior) were also measured (Figure 1(d)). These parameters were automatically measured using the AngioVue Imaging System (Optovue). Low-quality OCTA images with a scan quality <7/10 were excluded from the analysis. Central macular thickness (CRT) was determined as the mean retinal thickness in the central foveal region which was defined as the circular 1 mm diameter area centered on the fovea set by the Heidelberg Spectralis SD-OCT algorithm [17]. It was evaluated by two observers (BL and HW). Any result difference greater than 30% between graders was resolved by open discussion between the readers. If no consensus could be reached, they discussed the results with the senior (X.D.S) until the discrepancy was resolved.

The values of all continuous variables are presented as the mean plus or minus the standard deviation, and the categorical data are described as the frequency counts and percentages. All the quantitative data fit a normal distribution and a paired *t*-test was used to compare ocular parameters of RRD eyes after PPV with the fellow eyes. Possible associations between the macular or peripapillary VD and postoperative BCVA were analyzed by applying Pearson correlations. Multivariate linear regression analysis was performed to identify the factors associated with visual outcomes three months after RRD surgery. All statistical analyses were performed using SPSS software, version 20.0 (SPSS, IBM Inc.). *P* < 0.05 was considered statistically significant.

## 3. Results

Forty-four patients underwent PPV in our department for the repair of macula-off RRD between January 2018 and June 2019. Of these, 13 eyes were excluded for the presence of proliferative vitreoretinopathy grade C or worse (*n* = 2), vitreous hemorrhage (*n* = 1), glaucoma (*n* = 2), persistent subretinal fluid (*n* = 2) or intraretinal fluid (*n* = 1), or failure to attend regular follow-up visits (*n* = 5). In sum, 31 eyes with macula-off RRD were studied. Among them, three eyes (9.7%) were pseudophakic at baseline, and the remaining 28 eyes were phakic (90.3%).  Table 1 shows the demographic data of the patients with RRD. The mean age was 56.0 ± 9.5 years. The mean duration of symptoms was 3.6 ± 3.9 weeks. Eight eyes (25.8%) were filled with silicone oil and 23 eyes (74.2%) were filled with gas. LogMAR BCVA improved from 1.10 ± 0.50 before surgery to 0.58 ± 0.44 three months after vitrectomy. The neural retina reattached in all eyes after the initial surgery.

Table 2 demonstrates the postoperative ocular characteristics and OCTA characteristics in the macular region in comparison with the fellow eyes. There were no interocular differences in AL, refractive errors, or IOP. The mean FAZ areas in eyes with RRD three months after surgery and fellow eyes were 0.32 ± 0.13 mm^2^ and 0.32 ± 0.12 mm^2^, respectively. The superficial FFD (FFD-S) in eyes after RRD surgery and fellow eyes were 19.5 ± 7.3% and 16.8 ± 7.9%, respectively. The deep FFD (FFD-D) in eyes after RRD surgery and fellow eyes were 33.9 ± 8.8% and 31.7 ± 9.5%, respectively. There were no interocular differences in FAZ, FFD-S, superficial PFD (PFD-S), FFD-D, and deep PFD (PFD-D).

The optic nerve OCTA measurements in eyes after RRD surgery and the fellow eyes are displayed in Table 3. The total peripapillary VD was significantly reduced in eyes after RRD surgery (46.3 ± 5.6%) when compared with the fellow eyes (49.1 ± 7.0%) (*P* < 0.01). Likewise, eyes with RRD after surgery had a lower superior and inferior peripapillary VD compared with the fellow eyes (*P* < 0.01). However, there was no significant difference between the groups in ONH VD (*P*=0.495).

**Figure 2:**
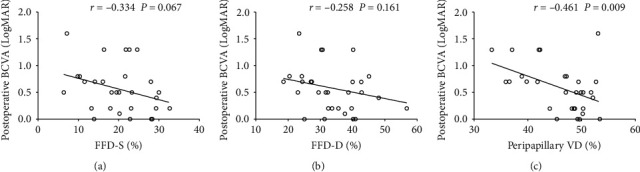
Scatterplots of associations between the postoperative (a) superficial foveal flow density (FFD-S), (b) deep foveal flow density (FFD-D), (c) peripapillary vessel density (VD), and postoperative best-corrected visual acuity (BCVA) three months after surgery.

Postoperative BCVA was correlated with FFD-S (*r* = −0.334, *P*=0.067), peripapillary VD (*r* = −0.461, *P*=0.009), baseline BCVA (*r* = 0.506, *P*=0.004), and selection of tamponade (*r* = 0.225, *P*=0.112) (Figure 2, Table 4). FFD-S, FFD-D, peripapillary VD, and baseline BCVA were included to perform a multivariate linear regression model to screen for the independent predictors of visual outcomes three months after RRD surgery. The results showed that patients with a higher peripapillary VD three months after PPV were more likely to achieve better postoperative BCVA [*P*=0.024, (95% CI: –0.054 to –0.004)]. Patients with better BCVA at baseline were also more likely to achieve better postoperative BCVA [*P*=0.012 (95% CI: 0.085–0.618)] (Table 4).

## 4. Discussion

The analysis of the macula and optic disc is essential for diagnosis and follow-up of retinal diseases as well as neuroophthalmic diseases. In this study, we evaluated the microvasculature changes in the macula and optic disc in RRD patients after PPV using OCTA. The results showed that the parafoveal retinal microvasculature in both the superficial and deeper layers of RRD patients after PPV surgery was close to that of the fellow eyes, while eyes after PPV surgery showed significant microvascular changes of the ONH. In addition, a higher level of peripapillary VD and SO tamponade were found to correlate with better postoperative BCVA. The present study may provide a key to understanding the link between perfusion of the macula and ONH in the context of RRD surgery.

Previous studies evaluating microvasculature after RRD focused on microvasculature changes in the macula. Our aim was to study the microvasculature changes in the macula and the optic disc. After exclusion of patients with abnormalities on OCT that may affect macular function, our data revealed that the FAZ area among RRD patients three months after PPV was similar to the fellow eyes. The superficial and deep parafoveal flow density was attenuated after PPV compared to the fellow eyes, though not a statistically significant difference. This was in consistent with previous studies by Woo et al. [9]. Yoon et al. also reported a similar finding after macular hole surgery [18]. One possible reason may be that both the superficial and the deep macular capillary plexus are vulnerable to hypoxia and increased inflammatory cytokine levels after RRD. Interestingly, we found that even though superficial FAZ recovered three months following RD repair, the capillary plexus did not return to the normal level, though subretinal fluid had completely disappeared. This was also consistent with the anatomical finding that eyes had a significant decrease in the thickness of all retinal layers after primary RD surgery [7].

The optic nerve OCTA showed that eyes after RRD surgeries had significantly decreased peripapillary VD compared to the fellow eyes. Both superior and inferior peripapillary VD in RRD eyes were lower than that of the fellow eyes. These findings were in line with recent studies on blood flow of the ONH showing that blood vessels on the ONH revealed significantly lower mean blur rate in eyes with RRD by laser speckle flowgraphy [19]. The radial peripapillary capillaries density in our study reflected most of the superficial layer and part of the deeper layers (the prelaminar and lamina cribrosa regions) of capillaries located around the optic disc. Three months after surgery, the ONH circulatory disorder might not recover though the retina reached successful anatomical reattachment and the macular capillary perfusion recovered close to the normal range. Henry et al. also reported that macular vessel density showed a relatively lower correlation with visual field severity than with peripapillary vessel density in glaucoma [20]. One possible reason might be that superficial retinal vessels in the parafoveal area are the terminal branches of retinal vessels. The vessel dropout may exhibit a stepwise decrease and reach a floor during the late stage of retinal detachment.

Our study also discovered that postoperative eyes with higher peripapillary VD had a better prognosis of visual outcomes. One possible explanation is that patients with RRD may develop rarefaction of retinal vessels, leading to ischemia and tissue hypoxia in both the macula and the ONH area. These pathological alterations may lead to persistent hypoperfusion in the ONH several months after the vitrectomy, the loss and permanent functional impairment of retinal ganglion cells and photoreceptors, and thus compromising visual acuity. These findings were similar to those reported in previous studies of patients with glaucoma that lower peripapillary VD was detected in early glaucoma [21] and was associated with the progression of glaucoma [22]. Since pars plana vitrectomy has been reported to increase the risk of open-angle glaucoma [23], it is of interest to investigate whether the decrease of peripapillary VD after vitrectomy in RRD patients is associated with the progression of glaucoma in long-term follow-up.

It was noteworthy that our study also found that SO tamponade showed a weaker correlation with better visual outcomes than gas tamponade. One possible explanation might be that the unintentional displacement of the retina after repair of macula-off RRD with PPV was seen more significantly in patients using gas than that using SO [24], which would cause postoperative visual disturbances. However, unexplained vision loss in eyes with good visual potential has been reported in RRD with SO tamponade [25–27]. One possible reason for this discrepancy might be that the mean tamponade duration in Eibenberger et al.'s study was about twelve months, which is much longer than our study. SO tamponade was associated with greater risk of elevated IOP and had long-term effects on IOP [28], and emulsified SO was reported to be toxic to the retina by entering the intraretinal space and causing retinal thinning [7]. The long-term effect of SO tamponade on VA and change of retinal blood flow need further investigation.

This study has several limitations. First, the sample size was relatively small and OCTA parameters were not measurable before the RRD surgery due to the elevation of neuroretina during RRD. Therefore, the fellow normal eyes were chosen as control. FAZ area is also strongly affected by the axial length of the eye. We have not corrected the influence of axial length in our study. Further studies with large samples are needed to elucidate the effects of axial length on FAZ. Second, there was no randomization for gas or SO tamponade. The choice of tamponade was determined by the surgeon on the basis of multiple factors, including the estimated risk of redetachment, grade of PVR, and size of retinal tear. However, the randomization of the choice of tamponade without grade of PVR would not be feasible in current practice. Third, it remains unknown which period following RD repair is most appropriate for evaluating the vascular changes in the macula and ONH. Relying on the longer time of SO tamponade would increase the risk of multiple complications like emulsification of SO and ocular hypertension; therefore, further investigation of long-term effect of SO tamponade on VA and change of retinal blood flow is necessary.

In conclusion, OCTA analyses revealed a significant decrease in peripapillary VD after pars plana vitrectomy in patients with RRD. A milder decrease in peripapillary VD, better BCVA at baseline, and choice of SO tamponade during RRD surgery were more likely to achieve better BCVA three months after pars plana vitrectomy. Further studies with a larger sample size and a longer observation period would be necessary to assess the practical utility of OCTA and confirm our findings.

## Figures and Tables

**Table 1:** Demographics characteristics for the study subjects.

	Eyes with RRD (*n* = 31)
*Age, years*	56.0 ± 9.5
Male/female, *n* (%)	13 (41.9)/18 (58.1)
Refractive error, *D*	−3.36 ± 3.90
Axial length, mm	24.8 ± 1.7
Duration of symptoms, weeks	3.6 ± 3.9

*IOP, mmHg*	
Baseline	15.5 ± 3.2
3 months	15.2 ± 3.1

*BCVA, LogMAR*	
Baseline	1.10 ± 0.50
3 months	0.58 ± 0.44

*Tamponade*	
Silicone oil, *n* (%)	8 (25.8)
Air, *n* (%)	23 (74.2)
SBP, mmHg	132.3 ± 16.7
DBP, mmHg	86.8 ± 8.6

IOP = intraocular pressure; BCVA = best-corrected visual acuity; SBP = systolic blood pressure; DBP = diastolic blood pressure.

**Table 2:** Macular characteristics in RRD eyes and fellow eyes using OCT and OCTA three months after PPV surgery.

	RRD eyes	Contralateral eyes	*P* value
Refractive errors, *D* (before surgery)	−3.36 ± 3.90	−2.76 ± 2.67	0.488^a^
Axial length, mm	24.8 ± 1.7	24.6 ± 1.0	0.621^a^
IOP, mmHg	15.2 ± 3.1	15.7 ± 2.8	0.471^a^
CRT	247.5 ± 51.3	256.5 ± 27.4	0.338^a^
FAZ	0.32 ± 0.13	0.32 ± 0.12	0.898^a^
Vascular density, FFD-S (%)	19.5 ± 7.3	16.8 ± 7.9	0.113^a^
Vascular density, PFD-S (%)	46.6 ± 5.7	48.5 ± 5.5	0.150^a^
Vascular density, FFD-D (%)	33.9 ± 8.8	31.7 ± 9.5	0.292^a^
Vascular density, PFD-D (%)	48.9 ± 4.4	50.1 ± 6.1	0.330^a^

CRT = central retinal thickness; FAZ = foveal avascular zone; S = superficial; FFD = foveal flow density; PFD = parafoveal flow density; D = deep; RRD = rhegmatogenous retinal detachment; PPV = pars plana vitrectomy. ^a^Paired sample *t*-test.

**Table 3:** Characteristics of ONH region in RRD eyes and fellow eyes using OCTA three months after PPV surgery.

	Eyes after RD surgery	Contralateral eyes	*P* value
ONH VD (%)	48.3 ± 8.3	49.2 ± 5.7	0.495^a^
Peripapillary VD (%)	46.3 ± 5.6	49.1 ± 7.0	0.003^a^
Superior peripapillary VD (%)	46.0 ± 6.0	49.0 ± 7.7	0.004^a^
Inferior peripapillary VD (%)	46.6 ± 6.7	49.3 ± 6.7	0.005^a^

ONH = optic nerve head; VD = vessel density; RRD = rhegmatogenous retinal detachment; PPV = pars plana vitrectomy. ^a^Paired sample *t*-test.

**Table 4:** Univariate and multivariate analysis of factors associated with postoperative BCVA.

Univariate factor analysis			Multivariable analysis			
Factors	Correlation coefficient	*P*	Factors	*β* coefficient	Multivariate adjusted OR (95% CI)	*P*
Baseline BCVA	0.506	0.004	Baseline BCVA	0.407	0.085 to 0.618	0.012
FFD-S	−0.334	0.067	FFD-S	−0.191	−0.041 to 0.018	0.426
FFD-D	−0.258	0.161	FFD-D	−0.028	−0.025 to 0.023	0.905
Peripapillary VD	−0.461	0.009	Peripapillary VD	−0.364	−0.054 to −0.004	0.024
Tamponade	0.225	0.112				

BCVA = best-corrected visual acuity; S = superficial; D = deep; FFD = foveal flow density; VD = vessel density; OR = odds ratio; CI = confidence interval.

## Data Availability

The data used to support the findings of this study are restricted by the Ethical Review Committee of Shanghai General Hospital, Shanghai Jiao Tong University School of Medicine, in order to protect patient privacy.
